# Mucosal Involvement in Bullous Pemphigoid Is Mostly Associated with Disease Severity and to Absence of Anti-BP230 Autoantibody

**DOI:** 10.3389/fimmu.2018.00479

**Published:** 2018-03-13

**Authors:** Ariane Clapé, Céline Muller, Grégory Gatouillat, Sébastien Le Jan, Coralie Barbe, Bach-Nga Pham, Frank Antonicelli, Philippe Bernard

**Affiliations:** ^1^Laboratory of Dermatology, Faculty of Medicine, EA7319, University of Reims Champagne-Ardenne, Reims, France; ^2^Department of Dermatology, Reims University Hospital, University of Reims Champagne-Ardenne, Reims, France; ^3^Laboratory of Immunology, Reims University Hospital, University of Reims Champagne-Ardenne, Reims, France; ^4^Clinical Research Unit, Reims University Hospital, Reims, France; ^5^Department of Biological Sciences, Immunology, UFR Odontology, University of Reims Champagne-Ardenne, Reims, France

**Keywords:** bullous pemphigoid, BP Disease Area Index, mucosal involvement, anti-BP230, autoantibodies

## Abstract

Bullous pemphigoid (BP) is the most common autoimmune bullous disease and typically affects the elderly. Binding of specific autoantibodies to BP180/230 hemidesmosomal components induces an inflammatory response leading to skin blister formation. Unusual manifestations of BP include additional mucous membrane involvement, without pathophysiological knowledge associated to the formation of these lesions. We here performed a prospective study on series of consecutive BP patients with (*n* = 77) and without (*n* = 18) mucosal involvements at baseline to further investigate why some BP patients display mucosal lesion and other not. Analysis of disease activity showed that BP patients with mucosal involvement displayed a higher total BP Disease Area Index (BPDAI) score (*P* = 0.008), but also higher skin and blister/erosion BPDAI scores (*P* = 0.02 and *P* = 0.001, respectively). By contrast, the erythema/urticaria BPDAI score was identical between the two groups of patients. The erythema/urticaria BPDAI score, but not the blister/erosion BPDAI score, was correlated with the serum concentration of anti-BP180 NC16A autoantibodies in patients with mucosal involvement. In multivariate analysis, the absence of anti-BP230 autoantibody was the only factor independently associated with mucosal involvement (OR 7.8; 95% CI, 3.1–19.6) (*P* < 0.0001). Analysis of the distribution of BP patients according to BPDAI scores revealed a shift toward higher blister/erosion BPDAI scores for BP patients with mucosal involvement. This study indicates that mucosal lesions are clinically mainly related to disease severity and immunologically to the absence of anti-BP230 antibodies.

## Introduction

Bullous pemphigoid (BP) is an autoimmune skin disease characterized by the binding of autoantibodies directed against two hemidesmosal proteins of the dermal–epidermal junction, namely BP180 and BP230 (1–6). The disease typically presents in the elderly with a generalized pruritic blistering eruption (7–9), which results from an inflammatory associated disruption of the dermal–epidermal junction induced by the binding of autoantibodies onto their targets. Clinical criteria for typical skin eruption of BP include the absence of associated external mucous membrane (almost exclusively oral) involvement, the absence of skin atrophy, and the absence of head and neck predominant involvement (10). However, clinical presentation of BP can be polymorphic, notably during the early, pre-bullous stage of the disease or in atypical variants, in which full-blown bullous lesions may be absent (11, 12). Beside, blisters and erosions arising on mucosal membranes, mainly within the oral cavity, may be observed in up to 20% of BP patients (6, 11–13), without identified pathophysiologic mechanism(s) associated with their development.

Despite the interest of the research community in always better understanding BP pathophysiology, no study demonstrated whether mucosal involvement occurred as a consequence of BP extent and severity or whether skin and mucosal lesions occurred concomitantly. Recently a clinical activity score named BP Disease Area Index (BPDAI) was proposed as an international consensus to evaluate both the disease extent and the location and type of skin lesions (9). The BPDAI also has the advantage to measure separate scores for skin and mucous membrane activity. Besides, the skin BPDAI score also evaluates separately the ultimate skin lesions, i.e., blisters/erosions, and the early, pre-bullous inflammatory skin lesions, i.e., urticaria/erythema, and their extent as well as the residual damages. Thus, such specific clinical score may be useful to better characterize those BP patients with associated mucosal involvement and improve their monitoring.

Immunological and biological investigations in BP brought strong evidence that autoantibodies to BP180 are pathogenic and play a key role in subepidermal blister formation (14–20). Biopsy specimens from BP lesional skin exhibit dense inflammatory infiltration of eosinophils, basophils, neutrophils, lymphocytes, and mast cells in the dermis (1, 11, 13, 21, 22). Inflammatory cells infiltration and activation release cytokines and proteases that may create an auto-amplification loop reported to induce dermal–epidermal separation (23–27). However, variations in this pathophysiological process are still missing to explain why some BP patients will exhibit mucosal involvement and other not. In this matter, a more precise clinical characterization of BP patients with mucosal involvement may help to point out variations in the autoimmune and inflammatory responses in this particular BP subset.

We here performed a prospective study on series of consecutive BP patients with and without mucosal involvement. To further understand why some BP patients display mucosal blisters or erosions and other not, we compared the total BPDAI and its different components with other clinical and immunological parameters of disease activity at baseline, including the number of daily new blisters and the anti-BP180 and anti-BP230 autoantibody titers.

## Materials and Methods

### Study Patients and Design

A prospective, single-center study was conducted in the Dermatology Department of the Reims University Hospital (French Referral Center for Autoimmune Bullous Diseases) between September 2013 and July 2017. The investigation was conducted under the approval of the Ethic Committee of the University Hospital of Reims (CNIL authorization DR-2013-320), and all of the subjects gave their informed and written consent before participating in the study in accordance with the Helsinki Declaration. Consecutive patients with newly diagnosed BP were included in this prospective study. Patients were diagnosed as having BP using the following criteria: clinical features typical of BP with the presence of at least three of four well-established criteria according to Vaillant et al. (10); subepidermal blister on skin biopsy, and deposits of IgG and/or C3 in a linear pattern along the epidermal basement membrane zone by direct immunofluorescence. Non-inclusion criteria were administration of a specific BP treatment for more than 2 days, pregnancy and expected survival after BP diagnosis shorter than 3 months.

### Clinical Data Collection

Clinical data recorded at baseline were gender, age, number of daily new blisters (determined over a 3-day period), and BPDAI. BPDAI was calculated according to the International Pemphigoid Committee recommendations (9). At baseline, BPDAI was recorded. The total BPDAI computes two scores: total BPDAI activity and total BPDAI damage. The total BPDAI activity score is the arithmetic sum of the three subcomponents—cutaneous blisters/erosions, cutaneous urticaria/erythema, and mucosal blisters/erosions. The total BPDAI damage score is the arithmetic sum of the items rated regionally for damage caused by more permanent features such as post-inflammatory hyperpigmentation, scarring, and other. BPDAI also takes into account lesion number and size thresholds and skin lesions are rated based on the regions affected. Scores can range from 0 to 360 for BPDAI total activity (maximum 240 for total skin activity and 120 for mucosal activity) and 0 to 12 for BPDAI damage, with higher scores indicating greater disease activity or damage. On the basis of previous literature (28), severe BP at baseline was defined as a total BPDAI score ≥56. BP patients who did not fulfill these criteria were classified as having moderate BP.

### Anti-BP180 and Anti-BP230 Autoantibody Detection

Anti-BP180 and anti-BP230 autoantibodies were detected in serum and in blister fluids (BFs) using specific commercially available enzyme-linked immunosorbent assays (ELISAs) following the manufacturer’s instructions (MBL, Nagoya, Japan). Anti-BP180 and anti-BP230 antibodies’ detection was routinely performed on classically used dilution with no further dilution when titers were high. ELISA values were expressed as units per milliliters of serum (U/mL), and the 9 U/mL cut-off value recommended by the manufacturer was used in both anti-BP180 and anti-BP230 ELISAs (29–32).

### Statistical Analysis

Quantitative variables were described as mean ± SD and qualitative data as number and percentage. Kolmogorov–Smirnov test was performed to compare BP population distribution. The Kolmogorov–Smirnov test works by comparing the cumulative distribution of the two data sets and computes a *P* value that depends on the largest discrepancy between distributions. In contrast to Mann–Whitney test that is mostly sensitive to changes in the median, the Kolmogorov–Smirnov test is sensitive to changes in the shape, spread, or median of the two distributions. Chi^2^ tests were used for comparison between qualitative variables and nonparametric, Mann–Whitney tests for comparison between quantitative values. Owing to absence of normal distribution, comparisons between groups were performed using Spearman’s correlation coefficient to explore the relationship between continuous variables (BPDAI with immunological parameters, immunological parameters in serum with those in BF). A *P* value <0.05 was considered statistically significant. Multivariate analyses were then performed using stepwise logistic regressions, with enter and removal limits set at 0.20 and factors significant at *P* = 0.20 included.

## Results

### Patient Characteristics

A total of 97 patients with newly diagnosed BP were included in this study, 79 had typical clinical manifestations of BP and 18 presented with mucosal lesions in additional to skin blisters or erosions. In those BP patients with mucosal involvement, indirect immunofluorescence on salt-split skin was either positive with labeling of the epidermal side of the cleavage or negative. Using the same technique, two patients with positive immunolabeling on the dermal side only were subsequently excluded from the group with typical clinical BP presentation.

The clinical characteristics of those 77 patients with typical BP features (81%, 51 women and 26 men) and of the 18 BP patients with mucosal involvement (19%, 11 women and 7 men) are shown in Table 1. In the 18 BP patients with unconventional mucosal involvement, oral mucosal lesions were observed in 17 cases and anogenital mucosae lesions in 1 case. Among the 17 patients with oral lesions, 1 patient also displayed anogenital mucosae involvement, and another 1 had concomitant pharyngeal mucosa lesions. In those 18 BP patients, neither conjunctival nor nasal mucosa involvement was observed. Occurrence of mucosal lesions was correlated neither with the gender (*P* = 0.78) nor with the age (*P* = 0.24) of patients.

**Table 1:** Clinical characteristics of patients with BP according to mucosal involvement.

	All BP patients	Patients without mucosal involvement	Patients with mucosal involvement	*P*value
Number of patients (*n*)	95	77	18	NA
Sex ratio (F/M)	1.88	1.96	1.57	0.68
Age, mean ± SD	81.7 ± 9.3	82.4 ± 8.9	78.7 ± 10.6	0.18
Patients with at least 10 daily new blisters,^a^ *n* (%)	45 (47.4)	32 (41.6)	13 (72.2)	0.02
Number of daily new blisters,^a^ mean ± SD	19.3 ± 29.8	17.6 ± 27.1	26.5 ± 39.4	0.07
BPDAI global score, mean ± SD	39.6 ± 27	35.8 ± 25.1	56.1 ± 29.2	0.008
Activity of total skin involvement, mean ± SD	38.1 ± 26	35.1 ± 25	51.0 ± 26.8	0.021
Blisters/erosions, mean ± SD	24.9 ± 17.6	22.1 ± 16.4	36.9 ± 17.6	0.001
Erythema/urticaria, mean ± SD	13.2 ± 13.6	13 ± 13.3	14.1 ± 15.1	0.91
Activity of mucosal involvement, mean ± SD	0.8 ± 2.4	0	4.0 ± 4.2	NA
Damage, mean ± SD	0.8 ± 2	0.7 ± 1.8	1.1 ± 2.6	0.78
Patients with severe disease according BPDAI,^b^ *n* (%)	24 (25)	16 (20.8)	8 (44.4)	0.04

*Subsets of BP patients with or without mucosal involvement were compared and P-value was obtained using Chi^2^ tests for comparison between qualitative variables and nonparametric, Mann–Whitney tests for comparison between quantitative values*.

*^a^Determined over a 3-day period*.

*^b^Determined by BPDAI global score ≥56*.

*NA, not applicable; BP, bullous pemphigoid; BPDAI, BP Disease Area Index*.

### Skin Disease Activity and Mucosal Involvement

At the time of diagnosis, the number of patients with at least 10 daily new blisters was significantly higher in BP patients with mucosal involvement (*P* = 0.02) compared with patients with typical BP (Table 1), whereas the number of daily new blisters only tended to be higher in BP patients with mucosal involvement (*P* = 0.07).

Total BPDAI mean score was 39.6 ± 27 at baseline in the whole BP population (Table 1). Based on a cut-off BPDAI value of 56 (see Materials and Methods), the percentage of BP patients with a severe disease was significantly lower in BP patients without mucosal involvement (20.8%) as compared to those with mucosal involvement (44.4%) (Table 1; *P* = 0.04). Deeper analysis showed that compared with BP patients with a typical disease, BP patients with mucosal involvement displayed a higher total BPDAI score (Table 1; *P* = 0.008), but also higher skin BPDAI and blister/erosion BPDAI scores (Table 1; *P* = 0.021 and *P* = 0.001, respectively). By contrast, the erythema/urticaria BPDAI score was not different between those two groups of patients (Table 1; *P* = 0.91).

**Figure 1:**
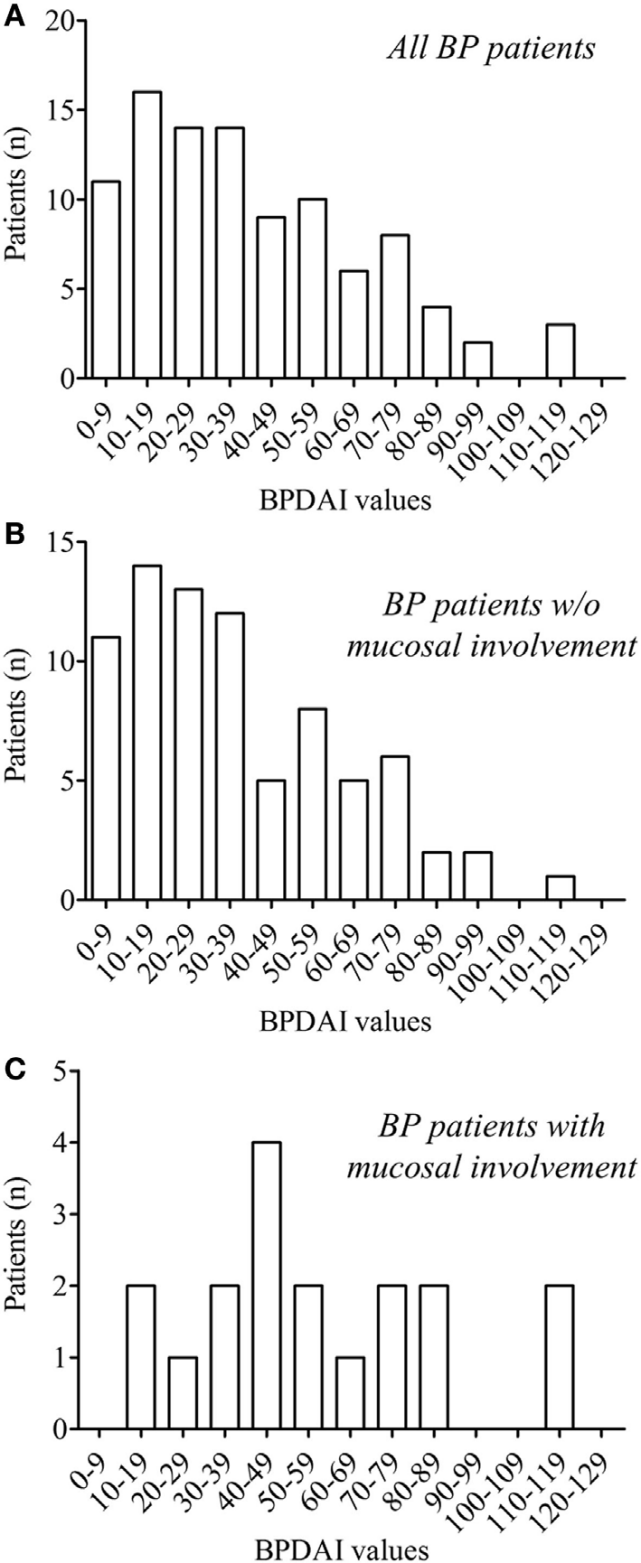
Distribution of the whole bullous pemphigoid (BP) patients (*n* = 95) **(A)** and of BP patients without (*n* = 77) **(B)** and with (*n* = 18) **(C)** mucosal involvement according to total BP Disease Area Index (BPDAI) values at the time of diagnosis. In the whole BP group and in the subgroup of BP without mucosal involvement, the number of BP included decrease when the values of the BPDAI increased, whereas BP patients with mucosal involvement were evenly distributed. Comparison of the cumulative distribution by the Kolmogorov–Smirnov test showed significant difference between BP patients with **(C)** and without **(B)** mucosal involvement (*D* = 0.6154; *P* = 0.008).

**Figure 2:**
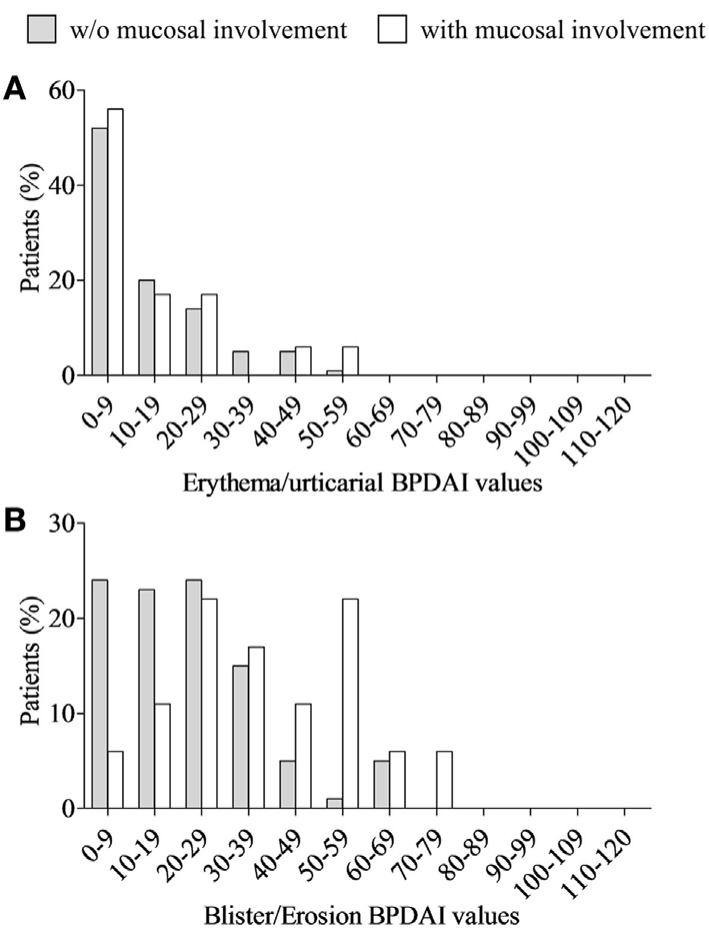
Distribution of both bullous pemphigoid (BP) patients with (white column) and without (gray column) mucosal involvement according to erythema/urticaria BP Disease Area Index (BPDAI) values **(A)** and blisters/erosions BPDAI values **(B)** at the time of diagnosis. No statistical differences were observed between the distributions of these two BP groups according to the Kolmogorov–Smirnov test, although a clear different distribution could be visualized within the low (0–19) and within the high (50–79) values of the blisters/erosions BPDAI values **(B)**.

Analysis of the whole BP population distribution according to BPDAI score revealed that most patients had a mild to moderate disease and that the higher the BPDAI was, the lower the number of BP patients was (Figure 1A). Such a distribution was also observed for BP patients without mucosal involvement (Figure 1B), but was significantly different in BP patients with mucosal involvement (*D* = 0.6154; *P* = 0.008). Indeed, BP patients with mucosal involvement were evenly distributed from moderate to severe disease (Figure 1C). To further understand these discrepancies, the same analysis was then performed using the separate skin activity. The relative distribution according to the erythema/urticaria BPDAI score of BP patients without mucosal involvement superposed with the one of BP patients with mucosal involvement (*D* = 0.1667; *P* = 0.991) (Figure 2A). Analysis of the distribution according to the blisters/erosions BPDAI revealed a high percentage of patients with mucosal involvement when the BPDAI values increased and a low percentage of those patients when the BPDAI values were low (*D* = 0.33; *P* = 0.433) (Figure 2B).

### Immunological Profiles and Mucosal Involvement

In the aim to analyze the clinical features through the BPDAI scores with regards to the autoimmune response, we first measured anti-BP180 and anti-BP230 autoantibody concentrations in the BF of patients with BP. Anti-BP180 and anti-BP230 antibodies were detected in the BF of 32 (86.5%) and 16 (43.2%) patients with a mean titer of 79.4 ± 51.2 and 31.2 ± 40.3 U/mL, respectively (Table 2A). Subgroup analysis of BP patients with and without mucosal involvement showed no significant difference both within the number of BP patients who produced these antibodies and within the mean BF titer related to each antibody. We then investigated whether serum autoantibody concentrations reflected those measured in the BF of the same patients to seek a relationship between BPDAI scores and autoantibody production in the whole BP patients included. Anti-BP180 and anti-BP230 antibodies titers within the BF were highly and significantly correlated with those measured in the serum (*r* = 0.78 and *r* = 0.73; *P* < 0.0001, respectively) (Table 2B). Such a correlation was also found for the anti-BP180 antibody titers within the two subsets of patients with or without mucosal involvement (*r* = 0.74 and *r* = 0.89; *P* < 0.0001 and *P* = 0.01, respectively) (Table 2B). Anti-BP230 antibody titers in BF from typical BP patients were highly and significantly correlated with those measured in the serum of these patients (*r* = 0.74; *P* < 0.0001), whereas only a high tendency was found for BP patients with mucosal involvement (*r* = 0.75; *P* = 0.07) (Table 2B).

**Table 2:** Blister fluid (BF) immunological characteristics (A) and correlation between serum and BF autoantibody titers (B) in patients with BP according to mucosal involvement.

	All BP patients	Patients without mucosal involvement	Patients with mucosal involvement	*P*value
**(A)**				

Number of patients (*n*)^a^	37	30	7	NA
BF anti-BP180 NC16A				
Number of patients with titer ≥9 U/mL, *n* (%)	32 (86.5)	26 (86.7)	6 (85.7)	0.95
Mean value ± SD (U/mL)	79.4 ± 51.2	78.5 ± 51.8	83.5 ± 52.2	0.66
BF anti-BP230				
Number of patients with titer ≥9 U/mL, *n* (%)	16 (43.2)	14 (46.7)	2 (28.7)	0.38
Mean value ± SD (U/mL)	31.2 ± 40.3	34.2 ± 41.5	18.2 ± 34.2	0.65

**(B)**				

Number of patients^a^	37	30	7	
Anti-BP180 NC16A	*r* = 0.78	*r* = 0.74	*r* = 0.89	
	*P* < 0.0001	*P* < 0.0001	*P* = 0.01	
Anti-BP230	*r* = 0.73	*r* = 0.74	*r* = 0.75	
	*P* < 0.0001	*P* < 0.0001	*P* = 0.07	

*Subsets of BP patients with or without mucosal involvement were compared and P-value was calculated using Chi^2^ tests for comparison between qualitative variables and nonparametric, Mann–Whitney tests for comparison between quantitative values. Spearman’s correlation coefficient was used to explore the relationship between immunological parameters in serum with those in BF*.

*^a^Patient for whom BF and serum anti-BP180 NC16A and anti-BP230 ELISA values were available*.

*NA, not applicable; BP, bullous pemphigoid; ELISA, enzyme-linked immunosorbent assay*.

**Figure 3:**
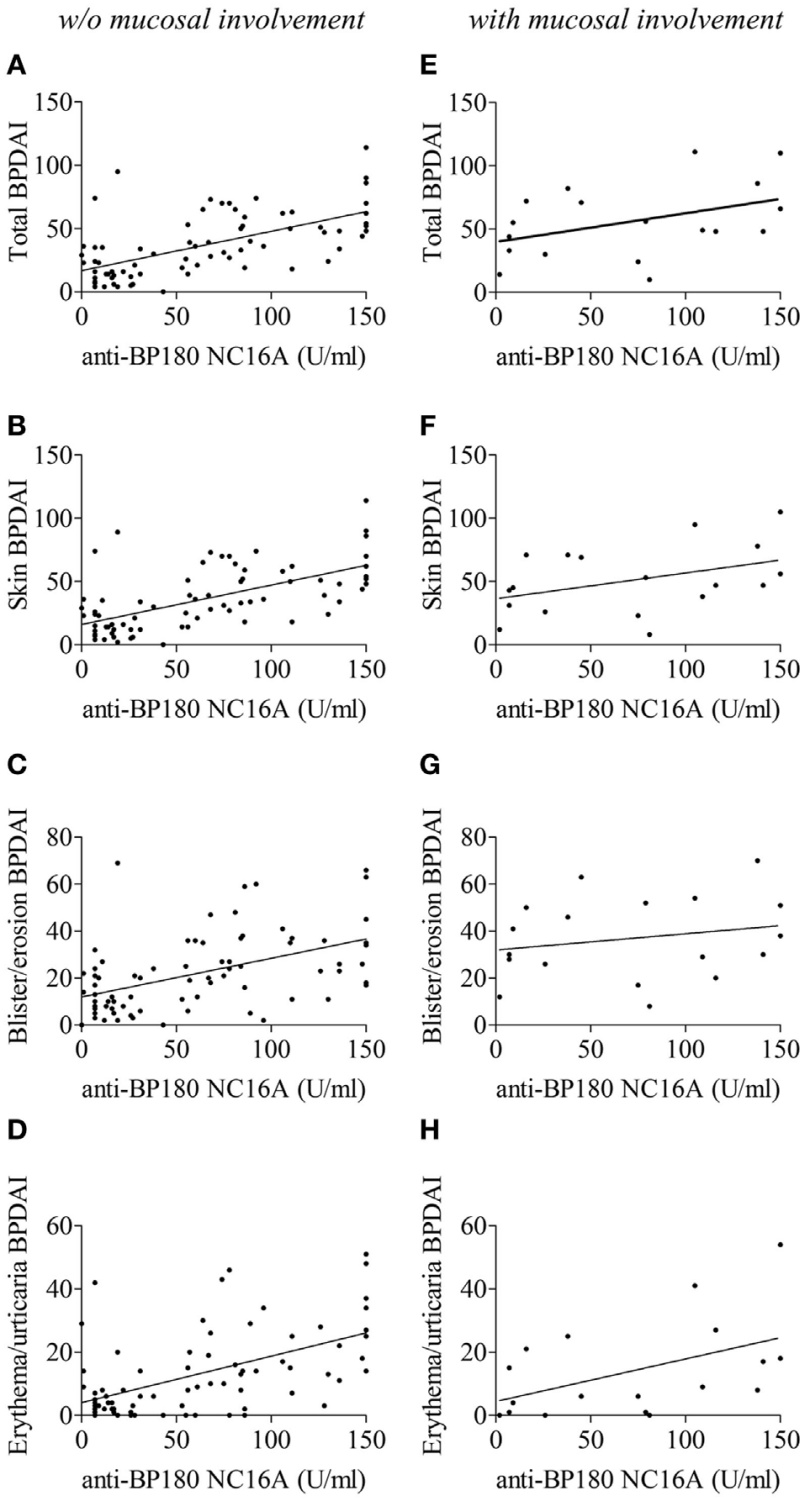
Correlation of serum anti-BP180 NC16A titers with total BP Disease Area Index (BPDAI) **(A,E)**, total skin BPDAI **(B,F)**, blister/erosion BPDAI **(C,G)**, and erythema/urticaria BPDAI **(D,H)** scores in bullous pemphigoid patients without **(A–D)** or with **(E–H)** mucosal involvement. The correlation coefficients and statistical significances were calculated according to Spearman’s correlation test and are summarized in Table 4.

Anti-BP180 antibodies were found in the serum of 77 patients (81.9%) with a mean titer of 63.9 ± 50.8 U/mL (Table 3). Subgroup analysis of BP patients with and without mucosal involvement showed no significant difference both within the number of BP patients who produced anti-BP180 antibodies and within the mean serum titer for this autoantibody (Table 3). In BP patients without mucosal involvement at baseline, serum anti-BP180 ELISA values were significantly correlated with all BPDAI scores except mucosal BPDAI, i.e., total, skin, blister/erosion, and erythema/urticaria BPDAI scores (*P* < 0.0001, Figures 3A–D, Table 4A).

**Table 3:** Serum immunological characteristics of patients with BP according to mucosal involvement.

	All BP patients	Patients without mucosal involvement	Patients with mucosal involvement	*P*value
**Serum anti-BP180 NC16A**				
Number of patients (*n*)^a^	94	76	18	NA
Number of patients with titer ≥9 U/mL, *n* (%)	77 (81.9)	62 (81.6)	15 (83.3)	0.86
Mean value ± SD (U/mL)	63.9 ± 50.8	62 ± 50.1	71.9 ± 54.4	0.53

**Serum anti-BP230**				
Number of patients (*n*)^b^	93	75	18	NA
Number of patients with titer ≥9 U/mL, *n* (%)	44 (47.3)	39 (52)	5 (27.8)	0.06
Mean value ± SD (U/mL)	29.1 ± 37	32.2 ± 37.8	16.2 ± 30.9	0.07

*Subsets of BP patients with or without mucosal involvement were compared and P-value was obtained using Chi^2^ tests for comparison between qualitative variables and nonparametric, Mann–Whitney tests for comparison between quantitative values*.

*^a^Patients for whom serum anti-BP180 ELISA values were available*.

*^b^Patients for whom serum anti-BP230 ELISA values were available*.

*NA, not applicable; BP, bullous pemphigoid; ELISA, enzyme-linked immunosorbent assay*.

**Table 4:** Correlation between BPDAI scores and serum anti-BP180 (A) or anti-BP230 (B) antibody titers in BP patients according to mucosal involvement.

	All BP patients	Patients without mucosal involvement	Patients with mucosal involvement
**(A)**			

Number of patients (*n*)^a^	94	76	18
Total BPDAI	*r* = 0.57	*r* = 0.62	*r* = 0.42
	*P* < 0.0001	*P* < 0.0001	*P* = 0.09
Total skin BPDAI	*r* = 0.58	*r* = 0.63	*r* = 0.46
	*P* < 0.0001	*P* < 0.0001	*P* = 0.06
Skin BPDAI: blisters/erosions	*r* = 0.46	*r* = 0.52	*r* = 0.26
	*P* < 0.0001	*P* < 0.0001	*P* = 0.30
Skin BPDAI: erythema/urticaria	*r* = 0.52	*r* = 0.53	*r* = 0.52
	*P* < 0.0001	*P* < 0.0001	*P* = 0.03
Mucosal BPDAI	*r* = 0.07	NA	*r* = 0.15
	*P* = 0.48	NA	*P* = 0.55

**(B)**			

Number of patients (*n*)^b^	93	75	18
Total BPDAI	*r* = −0.01	*r* = 0.05	*r* = 0.04
	*P* = 0.93	*P* = 0.69	*P* = 0.89
Total skin BPDAI	*r* = 0.001	*r* = 0.06	*r* = 0.02
	*P* = 0.99	*P* = 0.63	*P* = 0.94
Skin BPDAI: blisters/erosions	*r* = 0.03	*r* = 0.11	*r* = −0.05
	*P* = 0.76	*P* = 0.33	*P* = 0.86
Skin BPDAI: erythema/urticaria	*r* = 0.01	*r* = −0.01	*r* = 0.21
	*P* = 0.89	*P* = 0.9	*P* = 0.41
Mucosal BPDAI	*r* = −0.20	NA	*r* = −0.26
	*P* = 0.06	NA	*P* = 0.31

*Spearman’s correlation coefficient was used to explore the relationship between BPDAI score and serum autoantibody titers*.

*^a^Patients for whom serum anti-BP180 ELISA values were available*.

*^b^Patients for whom serum anti-BP230 ELISA values were available*.

*NA, not applicable; BP, bullous pemphigoid; BPDAI, BP Disease Area Index; ELISA, enzyme-linked immunosorbent assay*.

In BP patients with mucosal involvement at baseline, anti-BP180 ELISA values were only correlated with the erythema/urticaria BPDAI score (*P* = 0.03). Within this subgroup of BP patients, only a correlation tendency was observed with the total BPDAI score (*P* = 0.09) and the skin BPDAI score (*P* = 0.06), whereas no correlation could be drawn with the blister/erosion BPDAI score (*P* = 0.30) (Figures 3E–H, Table 4A). Finally, no correlation was found between the mucous membrane part of BPDAI score and the anti-BP180-NC16A ELISA values.

Anti-BP230 antibodies were found in the serum of 44 BP patients (47.3%) with a mean titer of 29.1 ± 37 U/mL (Table 3). Interestingly, 39 BP patients without mucosal involvement (52%) but only 5 among BP patients with mucosal involvement (27.8%) had a positive anti-BP230 ELISA value (*P* = 0.06). Such a difference tendency was also observed when analyzing the anti-BP230 mean titer (32.2 ± 37.8 and 16.2 ± 30.9 U/mL; *P* = 0.07, respectively) (Table 3). At baseline, anti-BP230 antibody serum concentrations were correlated with none of the BPDAI scores within these two groups (Table 4B).

### Factors Associated with Mucosal Involvement in BP

In univariate analysis, clinical features associated with mucosal involvement at baseline were a number of daily new blisters >10 (*P* = 0.02), a higher total, skin and blister/erosion BPDAI (*P* = 0.004, *P* = 0.02, *P* = 0.001, respectively), and a severe disease according BPDAI (*P* = 0.04). The absence of anti-BP230 autoantibody also showed a tendency of association with a mucosal involvement (*P* = 0.08). By contrast, univariate analysis revealed no relationship between anti-BP180 autoantibody serum concentration and mucosal involvement. In multivariate analysis, the absence of serum anti-BP230 autoantibody was the only factor independently associated with mucosal involvement (OR 7.8; 95% CI, 3.1–19.6) (*P* < 0.0001).

## Discussion

This study is the first one to compare at baseline the clinical characteristics by means of the BPDAI score along with the concentrations of anti-BP180 and anti-BP230 antibodies in BP patients with and without mucosal involvement. Analysis of separate and independent BPDAI sub-scores highlighted that skin lesions, more specifically blisters, and erosions were more substantial in patients with mucosal involvement than in typical BP. Such clinical characteristics were associated with the absence of anti-BP230 autoantibody in the serum of BP patients with mucosal involvement. Furthermore, we showed that mucosal lesions are clinically mainly related to disease severity, but not only. Altogether, our results suggest that compared to the classical pathophysiological processes previously defined in BP (12, 16, 17, 24, 33–36), specific immunological mechanisms should be triggered to end with mucosal lesions.

In BP patients with mucosal involvement, skin and mucosal lesions are likely to concomitantly occur during the disease process. Although our results showed that mucosal involvement in BP was associated with disease severity, we also observed mucosal involvement in BP patient with mild disease. Actually, there was a broad distribution of total BPDAI score among BP patients with mucosal involvement, but the percentage of patients with mucosal involvement increased along with the BPDAI values (Figure 1C). This suggests that mucous membrane involvement in patients with mild BP is not just a consequence of the extent or severity of the disease, and therefore that some BP patients are more prone to develop mucosal lesions than others. Thus, we can speculate that specific biological/immunological mechanisms responsible for these lesions are activated in those BP patients. This further raised the question of the event on which a break of tolerance outside of the skin tissue could originate. Occurrence of mucosal lesions in some patients with BP recalls the variation in the number of sites involved in patients with mucous membrane pemphigoid (37). Previous study suggested that regulatory T (T_reg_) cells play an indispensable role in maintaining self-tolerance in BP leading to an increase in autoreactive Th_2_, Th_1_, and B cells that can recognize different domains of BP180 (38–40). The role of T_reg_ cells in pemphigoid diseases was further demonstrated by mean of mice models (41). Although T_reg_ cells account for less than 10% of peripheral CD4+ T cells, recent studies have reported that tissue resident T_reg_ cells can even control non-immunological processes. Both T_reg_ types demonstrated plasticity and can convert to Th17 cells according to the cytokine environment (42). In this line, we and other groups demonstrated the presence of cytokines such as IL-1, IL-6, IL-23, TGFβ, etc, in BP skin lesions. We also demonstrated that the cytokines panel more likely originated from BP epiphenomenon that still remained to be elucidated (26). Such variations could account for variations in a deficit in T_reg_ cells and favor the development of tissue damages. In turn, skin remodeling could impact on both the number and the T_reg_ homing at different levels of the skin compartments. Although aging has been associated with a loss of central immune tolerance in elderly patients as in BP (43), further investigations are now required to determine the events specifically responsible for the mucosal involvement in patients with BP, especially of the oral cavity.

Although the analysis of skin BPDAI highlighted an increase in the blisters/erosions activity in BP patients with mucosal involvement, serum titers of autoantibodies directed against BP180-NC16A, the dominant autoantigen in BP, were not different between patients with and without mucosal involvement. Of note, Hofmann et al. showed that the presence of autoantibodies against both the BP180 N- and C-terminal of the ectodomain was associated with the presence of mucosal lesions (44). Then, Di Zenzo et al. (21) and Mariotti et al. (45) found that autoantibody reactivity against three extracellular epitopes of BP180 (NC16A, AA 1,080–1,107 and AA 1,331–1,404) appeared to be related to the presence of both skin and mucosal involvement in BP patients. By contrast, in our study, the uni- and multivariate analysis highlighted that the absence of anti-BP230 antibody was the only factor independently associated with mucosal involvement in BP. This unconventional result could imply that the immune response may be shifted toward other autoantibodies in those BP patients with mucosal involvement which could be related to a lower threshold of tolerance breakdown in line with a reduced level of T_reg_ cells or of T_reg_ homing as mentioned above. Based on a recent publication (20), we can hypothesize that the loss of immune tolerance curbs both the innate and the adaptive immune responses, which leads to the release of a specific panel of proteases associated with blister formation and tissue remodeling, and subsequently to a shift of the epitope targeted as compared with the classical pathophysiological mechanisms associated with BP. Altogether, this could be reflected by a reduced activity of the adaptive immune response toward the hemidesmosomal proteins of the basal keratinocyte layer and an increased activity toward the antigenic cryptic domains of molecules involved in the anchoring of those basal keratinocyte to the basal membrane structure. To address those mechanisms, it would be relevant to compare in future prospective studies the number and the activity of T_reg_ in biopsy specimens from either skin or mucosa BP lesions with others samples collected from patients with inflammatory non-autoimmune diseases.

The presence of other autoantibodies may interfere with the classical pathophysiological process of BP (12, 16, 17, 24, 33–36, 46). In BP patients without mucosal involvement, the serum concentration of anti-BP180 NC16A autoantibodies was well correlated with both the skin erythema/urticaria and the blisters/erosions BPDAI scores, which is in line with the fact that anti-BP180 autoantibody binding onto their target induced an inflammatory response leading to blister formation (14–18). By contrast, the correlation between anti-BP180 autoantibody titer and the skin blisters/erosions BPDAI score was not evidenced for BP patients with mucosal involvement. Of note, the BPDAI investigated the presence of both blisters and erosions. Thus, considering that the number of daily new blisters only displayed a higher tendency in BP patients with mucosal involvement, the highly significant difference in the BPDAI scores associated to blisters and erosions lesions may be related to the presence of erosions rather than to new blisters. This implies that the mechanisms leading to skin blisters/erosions and maybe to mucosal involvement too, rely on either different or additional trigger than anti-BP180-NC16A autoantibodies in BP patients with mucosal lesion. It was proposed that differential epitope recognition of BP180 could be associated with distinct clinical severity (5). However, additional studies are necessary to define the panel of autoantibodies associated with BP with mucosal lesions, to decipher the pathogenic role of these autoantibodies as well the immunologic mechanisms associated to their production.

Several limitations of the study are to be acknowledged, mainly its relatively limited number of BP patients with mucosal involvement. However, the prospective collection of clinical data including BPDAI assessment allowed a good evaluation of the percentage of BP patients with mucosal involvement, which was very close to results from previous large series of BP patients (5, 6, 21, 32, 44, 45) and from a recent retrospective study performed in our dermatology department (manuscript under consideration). Another weakness is the lack of investigations regarding biopsy specimens or BFs from oral mucous membrane for pathophysiological purpose. Unfortunately, recent intact oral blisters were rare and too transient for an effective, systematic sampling. To avoid biases, our present results were based on clinical or biological data which could systematically be collected in all consecutive patients. Although we observed that mucosal involvement also occurred in patients with mild or moderate disease, we here showed that mucosal involvement was more frequent in patients with severe BP according to the BPDAI score. Actually, in those severe BP patients, mucosal involvement could represent a severity criterion but not a clinical atypia. Considering all elements discussed above, further investigations should help defining the pathophysiological particularities of severe BP patients with mucosal involvement. Finally, in patients with moderate or minimal BP disease, other diagnoses could be considered, including anti-P200 pemphigoid and epidermolysis bullosa acquisita, which was very unlikely in our present series since indirect immunofluorescence on salt-split skin was either positive with labeling of the epidermal side of the cleavage or negative. The latter BP patients may represent an interesting subset of patients to identify in future studies the specific mechanisms responsible for mucous membrane lesions with the ultimate goal to propose innovative targeted therapy to preserve the quality of life for those elderly fragile patients.

## Ethics Statement

This study was carried out in accordance with the recommendations of the “Commission Nationale de l’Informatique et des libertés (CNIL, authorization DR-2013-320)” and approved by the Ethic Committee of the University Hospital of Reims. All subjects gave written informed consent in accordance with the Declaration of Helsinki.

## Author Contributions

FA, PB, and B-NP designed the study. AC, CM, GG, SJ, and CB performed experiments and analyzed clinical and biological data. AC, FA, and PB wrote the manuscript. All authors critically evaluated the data and approved the final version for publication.

## Conflict of Interest Statement

The authors declare that the research was conducted in the absence of any commercial or financial relationships that could be construed as a potential conflict of interest.
